# Prevalence of Gestational Diabetes Mellitus in Korea: A National Health Insurance Database Study

**DOI:** 10.1371/journal.pone.0153107

**Published:** 2016-04-05

**Authors:** Bo Kyung Koo, Joon Ho Lee, Jimin Kim, Eun Jin Jang, Chang-Hoon Lee

**Affiliations:** 1 Department of Internal Medicine, Seoul National University College of Medicine, Seoul National University Hospital, Seoul, Republic of Korea; 2 Department of Internal Medicine, Boramae Medical Center, Seoul, Republic of Korea; 3 Department of Obstetrics and Gynecology, Seoul National University College of Medicine, Seoul National University Hospital, Seoul, Republic of Korea; 4 National Evidence-Based Healthcare Collaborating Agency, Seoul, Republic of Korea; University of Tolima, COLOMBIA

## Abstract

**Aims/Introduction:**

This study aimed to estimate the prevalence of gestational diabetes mellitus (GDM) and use of anti-diabetic medications for patients with GDM in Korea, using data of the period 2007–2011 from the Health Insurance Review and Assessment (HIRA) database, which includes the claims data of 97% of the Korean population.

**Materials and Methods:**

We used the Healthcare Common Procedure Coding System codes provided by the HIRA to identify women with delivery in the HIRA database between 2009 and 2011. GDM was defined according to ICD-10 codes, and patients with pre-existing diabetes between January 1, 2007 and pregnancy were excluded. A Poisson regression was performed to evaluate the trends in annual prevalence rates.

**Results:**

The annual numbers of deliveries in 2009–2011 were 479,160 in 2009, 449,747 in 2010, and 377,374 in 2011. The prevalence of GDM during that period was 7.5% in 2009–2011: 5.7% in 2009, 7.8% in 2010, and 9.5% in 2011. The age-stratified analysis showed that the prevalence of GDM was highest in women aged 40–44 years, at 10.6% in 2009–2011, and that the annual prevalence significantly increased even in young women aged 20–29 years during that period (*P* < 0.05). More than 95% of the patients with GDM did not take any anti-diabetic medication. Among the anti-diabetic medications prescribed for patients with GDM, insulin was most commonly prescribed (for >98% of the patients with GDM on medication).

**Conclusions:**

The prevalence of GDM in Korean women recently reached 5.7–9.5% in recent years. This represents a public health concern that warrants proper screening and medical care for GDM in women during the childbearing years.

## Introduction

In the United States (U.S.), the prevalence of gestational diabetes mellitus (GDM) is increasing [1], and was approximately 9% in the period 2007–2010 [2], which is similar to the rates reported for other developed countries [3, 4]. With the changing prevalence of GDM, trends in prescription of glucose-lowering agents for patients with GDM have also changed. A retrospective cohort study of the nationwide population in the U.S., performed in 2000–2011, showed that glyburide replaced insulin as the most common pharmacotherapy for GDM [5], and glyburide use increased from 7.4% to 64.5% over the past decade [5].

Asian women have a comparable risk of GDM, despite having a relatively lower body mass index (BMI), compared to other ethnic groups [6, 7]. A community-based survey performed in the U.S. reported that the prevalence of GDM in Asian women was twice that of non-Hispanic white women [8]. In Asian populations, an increasing trend in GDM prevalence has been reported in Korea [9] and China [10]; however, the evidence is limited.

A history of GDM increases the subsequent risk of metabolic syndrome [11, 12], type 2 diabetes (T2D) [13], and subclinical atherosclerosis [14], even in young women. Therefore, proper screening and medical care for GDM in women during the childbearing years is likely to be important for public health. To support such an initiative, epidemiologic data regarding GDM should be collected and evaluated, including information about the management of patients with GDM.

The National Health Insurance (NHI) program in Korea provides universal health coverage, and the Health Insurance Review and Assessment (HIRA) service receives the claims of 97% of the population in Korea: in 2010, the number of beneficiaries from HIRA service was 48,906,795 among 50,515,666 the entire population of Korea [15]. The remaining 3% of the population are covered by the Medical Aid Program [15]. Accordingly, the HIRA database contains information on almost all of the insurance claims for approximately 51 million people living in Korea, including prescribed medications and procedures [16].

The present study aimed to determine the prevalence of GDM and use of medications during pregnancy for patients with GDM in South Korea, using data from the NHI claims database for 2007–2011. The Korean national guidelines during that period recommended all pregnant women to perform GDM screening at 24–28 weeks, regardless of underlying GDM risk [17]; therefore, the data on GDM prevalence calculated by using the claim data in Korea might be reliable for estimating the actual prevalence of GDM. We also compared the prevalence of comorbidities such as hypertension, polycystic ovary syndrome (PCOS), and prediabetes during pregnancy or prior to conception.

## Materials and Methods

We analyzed data from the HIRA database, which provides the following de-identified information on all insurance claims [16]: demographics, all medical services provided including the International Statistical Classification of Diseases and Related Health Problems, 10th edition (ICD-10) diagnostic code, and all medications dispensed. Values in key fields including drug name(s), quantity, date dispensed, and duration of prescriptions are missing or out of range in <0.5% of the records. This database has also been used in previous studies [18–21]. The study was approved by the Institutional Review Board of the Korean National Evidence-based Health Care Collaborating Agency.

We identified pregnant women with delivery between 2009 and 2011 using the codes from the Healthcare Common Procedure Coding System provided by the HIRA service (S1 Table). A pregnancy period was defined as 40 weeks before the date of delivery. Patients with GDM were identified based on the following criteria: (1) delivered a baby between 2009 and 2011, (2) had at least one claim under ICD-10 code O244 during a pregnancy period, and (3) did not have a claim for diabetes mellitus (ICD-10 codes E10–14) before pregnancy [22]. To exclude those with pre-existing diabetes before the study period, we excluded women who were diagnosed with any type of diabetes mellitus (E10–14) from January 1, 2007 until pregnancy. In the patients with GDM, glucose-lowering agents during the pregnancy period were identified. Claims for anti-hypertensive medications and any claims for glucose intolerance (ICD-10 code R730) or PCOS (ICD-10 code E282) during the 48 weeks before pregnancy were also analyzed. Pregnancy-induced hypertension (PIH) was identified using ICD-10 code O11-O15 during the pregnancy period.

Age-specific annual prevalence rates of GDM from 2009–2011 were calculated by dividing the number of patients with GDM by the entire sample of women with delivery during that period. A Poisson distribution was assumed to calculate 95% confidence intervals (CIs) for the prevalence rate of GDM, and Poisson regression was used to analyze the age-adjusted annual trends in prevalence rate. In addition, we compared the prevalence of hypertension, PCOS, and prediabetes prior to pregnancy using logistic regression analysis adjusted for age. All data were analyzed using SAS software version 9.3 (SAS Institute, Inc., Cary, NC, USA), and *P* < 0.05 was considered statistically significant.

## Results

The annual numbers of deliveries in 2009–2011 were 479,160 in 2009, 449,747 in 2010, and 377,374 in 2011, all of which were included in the final analysis. The prevalence of GDM during that period was 7.5% in 2009–2011: 5.7% in 2009, 7.8% in 2010, and 9.5% in 2011 (Table 1). After age-standardization of the data using the age structure of women with deliveries in 2010, the prevalence of GDM was similar: 5.8%, 7.8% and 9.5% in 2009, 2010, and 2011, respectively (S2 Table). Even in young women aged <30 years, GDM was detected in 5.2% during the entire study period (26,518 cases with GDM out of 511,652 deliveries). The prevalence of GDM was highest in women aged 40–44 years, at 10.6% in 2009–2011. When adjusted for age, the annual prevalence significantly increased from 2009 to 2011 (*P* < 0.001), and this trend of increasing GDM prevalence was identified in all age groups, except those aged <20 years (Table 1). Although no statistical significance was found in women aged <20 years, they also showed an increasing trend of the annual prevalence of GDM: 0.8% in 2009, 1.4% in 2010, and 1.8% in 2011.

**Table 1:** Prevalence rate of gestational diabetes mellitus (GDM) between 2009 and 2011.

Age (years)	2009			2010			2011			*P-*values
	Total pregnancies	Cases with GDM*	Prevalence, %	Total pregnancies	Cases with GDM*	Prevalence, %	Total pregnancies	Cases with GDM*	Prevalence, %	
Total	479,160	27,491	5.7	449,747	34,954	7.8	377,374	35,958	9.5	<0.001
<20	3,766	31	0.8	4,150	59	1.4	4,173	74	1.8	0.358
20–24	27,436	722	2.6	24,457	864	3.5	21,699	874	4.0	0.011
25–29	169,915	7,744	4.6	142,867	8,106	6.7	113,189	8,044	7.1	<0.001
30–34	204,210	13,157	6.4	202,138	18,144	9.0	169,532	18,485	10.9	<0.001
35–39	65,070	5,118	7.9	66,822	6,827	10.2	59,487	7,356	12.4	<0.001
40–44	8,202	704	8.6	8,743	926	10.5	8,769	1,095	12.5	<0.001
≥ 45	561	15	2.7	570	28	4.9	525	30	5.7	<0.001
<30	201,117	8,497	4.2	171,474	9,029	5.3	139,061	8,992	6.5	<0.001
≥ 30	278,043	18,994	6.8	278,273	25,925	9.3	238,313	26,966	11.3	<0.001

*Defined according to the following criteria: (1) underwent delivery during study period, (2) had at least one claim under ICD-10 code O244 during a pregnancy period and (3) did not have any claim of diabetes mellitus (under ICD-10 codes E10–14) before pregnancy

In the 48 weeks before the present pregnancy period, prediabetes and PCOS were detected in 3.0% and 1.7%, respectively, of the entire GDM sample. In addition, 8.1% of the patients with GDM were administered anti-hypertensive medications during the 48 weeks before the current pregnancy. The prevalence of prediabetes, PCOS, and prescription of anti-hypertensive medications before pregnancy in patients with GDM were significantly higher compared to cases without GDM even after adjustment for age (odds ratio [OR] = 1.529 [95% confidence interval [CI], 1.471–1.590, 1.321 [95% CI, 1.255–2.390], and 1.167 [95% CI, 1.471–1.590], respectively; Table 2). PIH during the current pregnancy period was also more frequently found in subjects with GDM compared to controls (OR = 3.561 [95% CI, 3.109–4.077]).

**Table 2:** Comorbidities during or prior to pregnancy.

	Case with GDM*(n = 98,403)		Case without GDM*(n = 1,207,878)					
	n	(%)	n	(%)	OR (95% CI)^‡^	*P*-value^‡^	OR (95% CI)^§^	*P*-value^§^
**Current pregnancy period**								
PIH during pregnancy period	6,054	(6.15)	40,024	(3.31)	3.779(3.302–4.325)	<0.0001	3.561(3.109–4.077)	<0.0001
**Prior 48 weeks before the present pregnancy**								
Diagnosis of impaired glucose metabolism (R730)	2,993	(3.04)	23,321	(1.93)	1.593(1.533–1.656)	<0.0001	1.529 (1.471–1.590)	<0.0001
Prescriptions of anti-hypertensive drug†	7,981	(8.11)	80,285	(6.65)	1.240(1.210–1.270)	<0.0001	1.167(1.139–1.196)	<0.0001
Diagnosis of PCOS (E282)	1,653	(1.68)	15,570	(1.29)	1.309(1.244–1.378)	<0.0001	1.321(1.255–1.390)	<0.0001

*Defined according to the following criteria: (1) underwent delivery during study period, (2) had at least one claim under ICD-10 code O244 during a pregnancy period and (3) did not have any claim of diabetes mellitus (under ICD-10 codes E10–14) before pregnancy

^†^Including beta-blocking agents, angiotensin-converting-enzyme inhibitor, angiotensin II receptor antagonists, diuretics, and calcium-channel blocking agents

^‡^Comparing the prevalence of comorbidities according to the presence and absence of GDM (without adjusting)

^§^Comparing the prevalence of comorbidities according to the presence and absence of GDM (with age-adjusting)

GDM, gestational diabetes mellitus; PIH, pregnancy induced hypertension; PCOS, polycystic ovary syndrome; OR, odds ratio; CI, confidence interval

Regarding glucose control during pregnancy, >95% of the patients with GDM were managed using medical nutritional therapy, without any glucose-lowering medication. Among the glucose–lowering agents prescribed for patients with GDM during pregnancy, insulin was prescribed the most often, for >98% of the patients with GDM on medication: 98.3% in 2009, 98.4% in 2010, and 98.4% in 2011 (Table 3). Metformin was prescribed for 0.4–0.7% of the entire sample of patients with GDM. Glyburide is not available in Korea; other sulfonylurea was prescribed for 0.1–0.2% of the entire sample of patients with GDM during the study period (Table 3).

**Table 3:** Glucose-lowering therapy during pregnancy in patients with gestational diabetes mellitus (GDM) between 2009 and 2011.

	2009		2010		2011	
	n	(%)	n	(%)	n	(%)
Medical nutrition therapy only	26,154	(95.14)	33,686	(96.37)	34,495	(95.93)
Insulin only	1,317	(4.79)	1,250	(3.58)	1,441	(4.01)
OAD only	9	(0.03)	8	(0.02)	11	(0.03)
Insulin + OAD	11	(0.04)	10	(0.03)	11	(0.03)
Insulin	1,328	(4.83)	1,260	(3.60)	1,452	(4.04)
Sulfonylurea	3	(0.01)	4	(0.01)	7	(0.02)
Meglitiniode	-	(0.00)	-	(0.00)	-	(0.00)
Metformin	18	(0.07)	15	(0.04)	17	(0.04)
Thiazoldinediones	-	(0.00)	1	(0.00)	-	(0.00)
DPP-4 inhibitor	1	(0.00)	1	(0.00)	-	(0.00)
α-glucosidase inhibitor	1	(0.00)	1	(0.00)	-	(0.00)

## Discussion

In this retrospective cohort study conducted in Korea using the NHI HIRA database, the age-adjusted annual prevalence of GDM in 2009–2011 was 7.5%. The year-specific prevalence was 5.7% in 2009, 7.8% in 2010 and 9.5% in 2011, which was similar to the previous reports in other Asian populations: 6.2–6.8% in 2007–2008 in China [10], and 9.7% in 2012–2013 in Bangladesh [23]. Recent population-based study from a city in China showed that the prevalence of GDM was 8.1% in 2010–2012, which is 3.5% increase compared to that in 1999 [24]. However, there have been a very limited number of studies on the prevalence of GDM in the other Asian countries in recent years.

Asian women have a relative high risk of GDM compared to Caucasians [6–8]. A community-based survey performed in the U.S. reported that the prevalence of GDM in Asian women was twice that of non-Hispanic white women [8], which reflects the importance of genetic factors in GDM, even after controlling for environmental factors. A genome-wide association analysis performed in Korean women showed that the genetic risk for GDM is associated with pancreatic β-cell function rather than insulin resistance [25]. The fact that the prevalence of obesity is lower in Korean women with GDM than in other populations [7], despite the known significant association between obesity and GDM [7, 26], also supports the relative importance of β-cell function in the Asian population for developing GDM.

Interestingly, despite a relatively short study period, we found that the prevalence of GDM significantly increased from 5.7% in 2009 to 9.5% in 2011, which is similar to the reports in the U.S. [2] and China [10, 24]. This increase in prevalence over a period of only a few years might be explained by recent changes in Korean statistics. For example, the maternal age is rapidly increasing, from an average age at first delivery of 29.3 years in 2003, 30.1 years in 2006, 31.3 years in 2012, to 31.5 years in 2013 [27]. Maternal age is one of the most important risk factors for GDM [1, 8]. In the present study, the prevalence of GDM increased with maternal age until 44 years of age, with a prevalence of 12% in 2011 in women aged 35–44 years, which was approximately twice that in women aged <40 years in the same year. Furthermore, as in the U.S. population [28], the number of plural pregnancies is also increasing in Korea: 12,062 in 2009, 12,841 in 2010, and 13,852 in 2011 [27]. Considering the association between the risk of GDM and increased placental mass and high human placental lactogen levels in pregnancy [29], it is possible that plural pregnancy is a risk factor for GDM.

However, the age-stratified analysis showed an increasing prevalence of GDM in Korea, even in young women aged 20–29 years, which is in contrast to the trend in the incidence of T2D in Korean women of the same age [20]. We recently reported a decreasing rate of incidence of diabetes mellitus from 2009–2011 in women aged 20–29 years using claim data: 1.0, 0.9, and 0.8 per 1,000 person-year in 2009, 2010, and 2011, respectively (*P* for trends <0.001) [20], which corresponded with the nationwide health survey in Korea [30] and in Japan [31]. Furthermore, BMI and waist circumference in young Korean women have significantly decreased during the 2000s [30]. Considering that GDM shares the risk factors with diabetes mellitus and obesity [7, 26], the increasing trends in the prevalence of GDM in our study should be interpreted with caution. Diagnostic criteria might influence the prevalence of disease in claim data. In Korea, a pregnant women are recommended to undergo GDM screening and diagnosis with a two-step approach using Carpenter-Coustan criteria or National Diabetes Data group (NDDG) criteria. In 2011, the Korean Diabetes Association (KDA) [32] adopted additionally a one-step approach according to the International Association of the Diabetes and Pregnancy Study Groups (IADPSG) criteria, which might explain the increasing prevalence of GDM, in part, as shown in other populations [33]. However, American College of Obstetricians and Gynecologists (ACOG) [34] recommends a two-step approach using Carpenter-Coustan criteria or NDDG criteria because there are no sufficient data on the superiority of IADPSG criteria as a GDM screening tool compared to a two-step approach, and only GDM screening via two-step approach can be covered by NHI in Korea; most obstetricians in primary care in Korea performed a two-step approach using Carpenter-Coustan criteria or NDDG criteria during the study period (between 2009 and 2011).

Regarding the use of anti-diabetic medication during pregnancy, the prescription rate and the most prevalent anti-diabetic medication in Korea are quite different from the U.S. First, this study showed that less than 5% of women with GDM took medication during pregnancy, which was lower compared to 8.1% from the claim data of the U.S. [5]. Second, more than 98% of the Korean patients with GDM in the present study were administered insulin, while glyburide has replaced insulin as the most common pharmacotherapy for GDM in the U.S. [5]. A body of evidence have shown that oral anti-diabetic medications including glyburide and metformin have the comparable efficacy to lower serum glucose level and no associated with the increases in the maternal and neonatal adverse outcomes, compared to insulin [35, 36]. However, recent studies such as the meta-analysis from randomized control studies [37], and retrospective cohort study from a nationwide U.S. insurance claims from 110, 879 women with GDM [38] showed that, the risks of neonatal hypoglycemia, high fetal birth weight, and macrosomia were higher in women with glyburide compared to insulin, although glyburide is as effective as insulin [37, 38]. In respect to other oral anti-diabetic agents, metformin showed a comparable maternal glycemic control and safety in neonatal hypoglycemia [36, 39]. Although preterm birth rate was significantly higher in metformin compared to insulin, metformin showed better maternal outcomes in terms of weight gain and pregnancy induced hypertension than insulin and better outcomes compared to glyburide in maternal weight gain and lower rates of macrosomia [39]; and it is notable that in women with PCOS, metformin treatment decreased the rate of preterm birth [40]. Glyburide and metformin are currently classified as Category C and B by the U.S. Food and Drug Administration (FDA) for use in pregnancy, respectively [36]; most practitioners in Korea are also reluctant to prescribe oral anti-diabetic agents, which might result in that almost pregnant women with GDM requiring anti-diabetic medications have been treated with insulin in Korea. However, considering that oral agents are less expensive, easier to administer, and demonstrate improved patient compliance as compared to insulin, oral anti-diabetic medication during pregnancy could be used for selected patients with GDM. To determine which patients would be appropriate candidates, selection criteria should be developed based on additional epidemiological data for Korean women.

As in a previous report [26], there was a relatively high prevalence of hypertension during the pre-pregnancy period in patients with GDM, as indicated by the use of anti-hypertensive medications in 8.1% of patients with GDM during the 48 weeks before pregnancy; this is significantly higher than the 6.6% in patients without GDM. The results of the present study support the careful evaluation of glucose tolerance status, even at the initial prenatal visit, in women with hypertension as suggested by the American Diabetes Association guideline [41]. The prevalence of PCOS and prediabetes in patients with GDM in our study were 1.68% and 3.04%, respectively, and were also significantly higher than those in women without GDM; however, these prevalence rates were considerably lower than those reported by previous epidemiological studies in Korea [42, 43], which might be due to the limitation of claims data.

We estimated the current prevalence of GDM in Korean women using data from nationwide health insurance claims in the HIRA database. Considering there is a very limited number of studies on the prevalence of GDM in Asian countries in recent years, especially in Korea, this extensive investigation of epidemiological data from a nationwide claims database might be helpful for planning national public health strategies. However, the study has certain limitations. First, the diagnosis of GDM was based on claims data, and discrepancies between the claims data and a true diagnosis could exist. We did not access the oral glucose tolerance test (OGTT) data of pregnancy women. Furthermore, as we could not identify maternal glycemic control level from claim data, the effectiveness of each glucose-lowering modality could not be evaluated. Although women with GDM taking glucose-lowering medications in Korea were no more than half of those in U.S., the present study could not assess the attainment of glucose control target in Korean women with GDM without medication. In addition, considering that the prevalence of PCOS and prediabetes in GDM women in the present study was lower than those reported by previous epidemiological studies in Korea, claims data also provide limited information on co-morbid conditions. Second, the proportion of the pregnant population who undergoes GDM screening can influence the prevalence of GDM in a claims-based study. We did not assess the rate of GDM screening in all of the delivery cases. However, Korean national guidelines recommended all pregnant women to perform GDM screening regardless of underlying GDM risk. Furthermore, because of NHI coverage, almost all of the pregnant Korean women undergo GDM screening during pregnancy. Third, we have no exact statistics on the method of GDM screening during the study period; the use of IADPSG criteria in the diagnosis of GDM is reported to be associated with an increased incidence of GDM [33]. However, as most obstetricians in primary care in Korea performed a two-step approach following ACOG guidelines and NHI coverage qualification, the change in the GDM screening and diagnosis method was not likely to have an influence on the prevalence of GDM over the study period. Lastly, a 3-year follow-up is relatively short to evaluate trends in the prevalence of GDM. However, we extensively reviewed at least 64 weeks of prior claims data to exclude pre-existing diabetes mellitus in the calculation of GDM prevalence, which could result in reasonable prevalence rates of GDM in Korea.

In conclusion, the prevalence of GDM in Korean women recently reached 5.7–9.5%, which is now comparable to that of developed countries. This extensive investigation of epidemiological data from a nationwide claims database will likely be invaluable for planning national public health strategies.

## Supporting Information

S1 Table Definition of delivery using Healthcare Common Procedure Coding System codes provided by HIRA. (DOCX)

S2 Table Prevalence of GDM after age-standardization using the Korean Women with delivery in the year 2010 as the standard. (DOCX)

## Abbreviations

GDM: gestational diabetes mellitus
NHI: the National Health Insurance
HIRA: Health Insurance Review and Assessment
ICD-10: International Statistical Classification of Diseases and Related Health Problems, 10th edition
PCOS: polycystic ovary syndrome
T2D: type 2 diabetes
PIH: pregnancy-induced hypertension
KDA: Korean Diabetes Association
KSOG: Korean Society of Obstetrics and Gynecology
IADPSG: International Association of the Diabetes and Pregnancy Study Groups
ACOG: American College of Obstetricians and Gynecologists
FDA: Food and Drug Administration
BMI: body mass index
U.S.: United States
CI: confidence intervals
OR: odds ratio

## References

[1] Ferrara A. Increasing prevalence of gestational diabetes mellitus: a public health perspective. Diabetes Care. 2007;30 Suppl 2:S141–6. 10.2337/dc07-s206 . 17596462

[2] DeSisto CL, Kim SY, Sharma AJ. Prevalence estimates of gestational diabetes mellitus in the United States, Pregnancy Risk Assessment Monitoring System (PRAMS), 2007–2010. Prev Chronic Dis. 2014;11:E104 10.5888/pcd11.130415 24945238 PMC4068111

[3] Lamberg S, Raitanen J, Rissanen P, Luoto R. Prevalence and regional differences of gestational diabetes mellitus and oral glucose tolerance tests in Finland. Eur J Public Health. 2012;22(2):278–80. 10.1093/eurpub/ckq193 . 21186187

[4] Jenum AK, Morkrid K, Sletner L, Vangen S, Torper JL, Nakstad B, et al Impact of ethnicity on gestational diabetes identified with the WHO and the modified International Association of Diabetes and Pregnancy Study Groups criteria: a population-based cohort study. Eur J Endocrinol. 2012;166(2):317–24. 10.1530/EJE-11-0866 22108914 PMC3260695

[5] Castillo WC, Boggess K, Sturmer T, Brookhart MA, Benjamin DK, Funk MJ. Trends in Glyburide Compared With Insulin Use for Gestational Diabetes Treatment in the United States, 2000–2011. Obstetrics and Gynecology. 2014;123(6):1177–84. WOS:000336809600006. 24807336 10.1097/AOG.0000000000000285 PMC4075168

[6] Wong VW. Gestational diabetes mellitus in five ethnic groups: a comparison of their clinical characteristics. Diabet Med. 2012;29(3):366–71. 10.1111/j.1464-5491.2011.03439.x . 21913963

[7] Choi SK, Park IY, Shin JC. The effects of pre-pregnancy body mass index and gestational weight gain on perinatal outcomes in Korean women: a retrospective cohort study. Reproductive biology and endocrinology: RB&E. 2011;9:6 10.1186/1477-7827-9-6 21241516 PMC3033321

[8] Ferrara A, Kahn HS, Quesenberry CP, Riley C, Hedderson MM. An increase in the incidence of gestational diabetes mellitus: Northern California, 1991–2000. Obstet Gynecol. 2004;103(3):526–33. . 14990417 10.1097/01.AOG.0000113623.18286.20

[9] Increasing prevalence of gestational diabetes in Korea from 2003 to 2012 [Internet]. Sejong: Ministry of Health and Welfare; 2014

[10] Zhang F, Dong L, Zhang CP, Li B, Wen J, Gao W, et al Increasing prevalence of gestational diabetes mellitus in Chinese women from 1999 to 2008. Diabet Med. 2011;28(6):652–7. 10.1111/j.1464-5491.2010.03205.x . 21569085

[11] Noctor E, Crowe C, Carmody LA, Kirwan B, O'Dea A, Glynn LG, et al ATLANTIC-DIP: prevalence of metabolic syndrome and insulin resistance in women with previous gestational diabetes mellitus by International Association of Diabetes in Pregnancy Study Groups criteria. Acta Diabetol. 2014 10.1007/s00592-014-0621-z . 25002067

[12] Lauenborg J, Mathiesen E, Hansen T, Glumer C, Jorgensen T, Borch-Johnsen K, et al The prevalence of the metabolic syndrome in a danish population of women with previous gestational diabetes mellitus is three-fold higher than in the general population. J Clin Endocrinol Metab. 2005;90(7):4004–10. 10.1210/jc.2004-1713 . 15840755

[13] Jang HC. Gestational diabetes in Korea: incidence and risk factors of diabetes in women with previous gestational diabetes. Diabetes Metab J. 2011;35(1):1–7. 10.4093/dmj.2011.35.1.1 21537406 PMC3080571

[14] Freire CM, Barbosa FB, de Almeida MC, Miranda PA, Barbosa MM, Nogueira AI, et al Previous gestational diabetes is independently associated with increased carotid intima-media thickness, similarly to metabolic syndrome–a case control study. Cardiovasc Diabetol. 2012;11:59 10.1186/1475-2840-11-59 22651701 PMC3403942

[15] Kang YK, Kim JD. 2010 Health Insurance Statistical Yearbook National Health Insurance Corporation and the Health Insurance Review and Assessment Service; 2011.

[16] Choi NK, Chang Y, Choi YK, Hahn S, Park BJ. Signal detection of rosuvastatin compared to other statins: data-mining study using national health insurance claims database. Pharmacoepidemiol Drug Saf. 2010;19(3):238–46. 10.1002/pds.1902 . 20049851

[17] KoreanDiabetesAssociation. Treatment guideline for diabetes. 3 ed: Korean Diabetes Association; 2007.

[18] Lee CH, Kim K, Hyun MK, Jang EJ, Lee NR, Yim JJ. Use of inhaled corticosteroids and the risk of tuberculosis. Thorax. 2013;68(12):1105–13. 10.1136/thoraxjnl-2012-203175 . 23749841

[19] Lee CH, Hyun MK, Jang EJ, Lee NR, Kim K, Yim JJ. Inhaled corticosteroid use and risks of lung cancer and laryngeal cancer. Respir Med. 2013;107(8):1222–33. 10.1016/j.rmed.2012.12.002 . 23768737

[20] Koo BK, Lee CH, Yang BR, Hwang SS, Choi NK. The incidence and prevalence of diabetes mellitus and related atherosclerotic complications in Korea: a National Health Insurance Database Study. PLoS One. 2014;9(10):e110650 10.1371/journal.pone.0110650 25329714 PMC4199756

[21] Kang YA, Choi NK, Seong JM, Heo EY, Koo BK, Hwang SS, et al The effects of statin use on the development of tuberculosis among patients with diabetes mellitus. The international journal of tuberculosis and lung disease: the official journal of the International Union against Tuberculosis and Lung Disease. 2014;18(6):717–24. 10.5588/ijtld.13.0854 . 24903944

[22] Sloan FA, Belsky D, Ruiz D Jr., Lee P. Changes in incidence of diabetes mellitus-related eye disease among US elderly persons, 1994–2005. Arch Ophthalmol. 2008;126(11):1548–53. Epub 2008/11/13. 10.1001/archopht.126.11.1548 126/11/1548 [pii]. 19001223 PMC3578294

[23] Jesmin S, Akter S, Akashi H, Al-Mamun A, Rahman MA, Islam MM, et al Screening for gestational diabetes mellitus and its prevalence in Bangladesh. Diabetes Res Clin Pract. 2014;103(1):57–62. 10.1016/j.diabres.2013.11.024 . 24369985

[24] Leng J, Shao P, Zhang C, Tian H, Zhang F, Zhang S, et al Prevalence of gestational diabetes mellitus and its risk factors in Chinese pregnant women: a prospective population-based study in Tianjin, China. PLoS One. 2015;10(3):e0121029 10.1371/journal.pone.0121029 25799433 PMC4370728

[25] Kwak SH, Kim SH, Cho YM, Go MJ, Cho YS, Choi SH, et al A genome-wide association study of gestational diabetes mellitus in Korean women. Diabetes. 2012;61(2):531–41. 10.2337/db11-1034 22233651 PMC3266417

[26] Campbell SK, Lynch J, Esterman A, McDermott R. Pre-pregnancy predictors of diabetes in pregnancy among Aboriginal and Torres Strait Islander women in North Queensland, Australia. Maternal and child health journal. 2012;16(6):1284–92. 10.1007/s10995-011-0889-3 . 21959925

[27] Kim YS. Live births by age group of mother, sex and birth order. In: Korea S, editor. Daejeon: Statistics Korea; 2014.

[28] Jewell SE, Yip R. Increasing trends in plural births in the United States. Obstet Gynecol. 1995;85(2):229–32. . 7824236 10.1016/0029-7844(94)00354-g

[29] Spellacy WN, Buhi WC, Birk SA. Human placental lactogen levels in multiple pregnancies. Obstet Gynecol. 1978;52(2):210–2. . 683661

[30] Koo BK, Kim EK, Choi H, Park KS, Moon MK. Decreasing trends of the prevalence of diabetes and obesity in korean women aged 30–59 years over the past decade: results from the korean national health and nutrition examination survey, 2001–2010. Diabetes Care. 2013;36(7):e95–6. Epub 2013/06/27. 10.2337/dc13-0247 36/7/e95 [pii]. . 23801818 PMC3687288

[31] Charvat H, Goto A, Goto M, Inoue M, Heianza Y, Arase Y, et al Impact of population aging on trends in diabetes prevalence: A meta-regression analysis of 160,000 Japanese adults. Journal of diabetes investigation. 2015;6(5):533–42. 10.1111/jdi.12333 26417410 PMC4578492

[32] KoreanDiabetesAssociation. Treatment guideline for diabetes. 4 ed Seoul: Korean Diabetes Association; 2011.

[33] Mayo K, Melamed N, Vandenberghe H, Berger H. The impact of adoption of the international association of diabetes in pregnancy study group criteria for the screening and diagnosis of gestational diabetes. Am J Obstet Gynecol. 2015;212(2):224 e1–9. 10.1016/j.ajog.2014.08.027 . 25173183

[34] Committee on Practice B-O. Practice Bulletin No. 137: Gestational diabetes mellitus. Obstet Gynecol. 2013;122(2 Pt 1):406–16. . 23969827 10.1097/01.AOG.0000433006.09219.f1

[35] Langer O, Conway DL, Berkus MD, Xenakis EM, Gonzales O. A comparison of glyburide and insulin in women with gestational diabetes mellitus. N Engl J Med. 2000;343(16):1134–8. 10.1056/NEJM200010193431601 . 11036118

[36] Nicholson W, Bolen S, Witkop CT, Neale D, Wilson L, Bass E. Benefits and risks of oral diabetes agents compared with insulin in women with gestational diabetes: a systematic review. Obstet Gynecol. 2009;113(1):193–205. . 19104375 10.1097/AOG.0b013e318190a459

[37] Zeng YC, Li MJ, Chen Y, Jiang L, Wang SM, Mo XL, et al The use of glyburide in the management of gestational diabetes mellitus: a meta-analysis. Adv Med Sci. 2014;59(1):95–101. 10.1016/j.advms.2014.03.001 . 24797983

[38] Camelo Castillo W, Boggess K, Sturmer T, Brookhart MA, Benjamin DK Jr., Jonsson Funk M. Association of Adverse Pregnancy Outcomes With Glyburide vs Insulin in Women With Gestational Diabetes. JAMA pediatrics. 2015;169(5):452–8. 10.1001/jamapediatrics.2015.74 . 25822253

[39] Balsells M, Garcia-Patterson A, Sola I, Roque M, Gich I, Corcoy R. Glibenclamide, metformin, and insulin for the treatment of gestational diabetes: a systematic review and meta-analysis. BMJ. 2015;350:h102 10.1136/bmj.h102 25609400 PMC4301599

[40] Vanky E, DEZ F, Diaz M, Ibanez L, Carlsen SM. On the potential of metformin to prevent preterm delivery in women with polycystic ovary syndrome–an epi-analysis. Acta obstetricia et gynecologica Scandinavica. 2012;91(12):1460–4. 10.1111/aogs.12015 . 23006146

[41] American Diabetes A. (1) Strategies for improving care. Diabetes Care. 2015;38 Suppl:S5–7. 10.2337/dc15-S004 . 25537709

[42] Kim CH, Kim HK, Kim BY, Jung CH, Mok JO, Kang SK. Impact of hemoglobin A1c-based criterion on diagnosis of prediabetes: The Korea National Health and Nutrition Examination Survey 2011. Journal of diabetes investigation. 2015;6(1):51–5. 10.1111/jdi.12245 25621133 PMC4296703

[43] Byun EK, Kim HJ, Oh JY, Hong YS, Sung YA. The prevalence of polycystic ovary syndrome in college students from Seoul. J Korean Soc Endocrinol. 2005;20:120–6.

