# Correction: Prevalence of Gestational Diabetes Mellitus in Korea: A National Health Insurance Database Study

**DOI:** 10.1371/journal.pone.0165445

**Published:** 2016-10-20

**Authors:** Bo Kyung Koo, Joon Ho Lee, Jimin Kim, Eun Jin Jang, Chang-Hoon Lee

The second affiliation for the fourth author was incorrectly omitted. Eun Jin Jang is affiliated with both National Evidence-Based Healthcare Collaborating Agency, Seoul, Republic of Korea, and Department of Information Statistics, College of Natural Science, Andong National University, Andong, Republic of Korea.
