# Supplementary material for: Prevalence of Gestational Diabetes Mellitus in Korea: A National Health Insurance Database Study
Source: PLoS One. 2016 Apr 5;11(4):e0153107. doi: 10.1371/journal.pone.0153107 (PMC4821493; doi:10.1371/journal.pone.0153107)
Supplement: S1 Table — (DOCX) [file pone.0153107.s001.docx]

Table S1. Definition of delivery using Healthcare Common Procedure Coding System codes provided by HIRA

| Mode of delivery | Code in Healthcare Common Procedure |
| --- | --- |
| Normal vaginal delivery | R4351, R4353, R4356, R4358, R4380 |
| Vaginal delivery using induction | R3131, R3133, R3136, R3138 |
| Vaginal delivery using vacuum | R3141, R3143, R3146, R3148 |
| Breech delivery | R4161, R4162 |
| Cesarean section delivery | R4517, R4518, R4514, R4519, R4520, R4516 |
