# Supplementary material for: Prevalence of Gestational Diabetes Mellitus in Korea: A National Health Insurance Database Study
Source: PLoS One. 2016 Apr 5;11(4):e0153107. doi: 10.1371/journal.pone.0153107 (PMC4821493; doi:10.1371/journal.pone.0153107)
Supplement: S2 Table — (DOCX) [file pone.0153107.s002.docx]

Table S2. Prevalence of GDM after age-standardization using the Korean Women with delivery in the year 2010 as the standard

| Age | 2009 | | | 2010 | | | 2011 | | |
| --- | --- | --- | --- | --- | --- | --- | --- | --- | --- |
|  | Total pregnancies | Crude Prevalence, % | Standardized Prevalence, % | Total pregnancies | Crude Prevalence, % | Standardized Prevalence, % | Total pregnancies | Crude Prevalence, % | Standardized Prevalence, % |
| Total | 479,160 | 5.74 | 5.83 | 449,747 | 7.77 | 7.77 | 377,374 | 9.53 | 9.48 |
| <30 years | 201,117 | 4.22 | 4.22 | 171,474 | 5.26 | 5.26 | 139,061 | 6.47 | 6.47 |
| ≥30 years | 278,043 | 6.83 | 6.83 | 278,273 | 9.31 | 9.31 | 238,313 | 11.31 | 11.32 |
